# Computational Modelling of Protected and Unprotected Head Impacts in Rugby

**DOI:** 10.3390/bioengineering12040361

**Published:** 2025-03-31

**Authors:** Thea Hodges, Adam Jones, Lucía Pérez del Olmo, Ashwin Mishra, Brian Caulfield, Tahar Kechadi, David MacManus, Michael D. Gilchrist

**Affiliations:** 1 School of Mechanical and Materials Engineering, University College Dublin, Belfield, D04 V1W8 Dublin, Ireland; 2 School of Public Health, Physiotherapy & Sports Science, University College Dublin, Belfield, D04 V1W8 Dublin, Ireland; 3 School of Computer Science, University College Dublin, Belfield, D04 V1W8 Dublin, Ireland

**Keywords:** sport biomechanics, head impact, concussion, video reconstruction, instrumented mouthguard, impact monitoring mouthguard

## Abstract

This study involved the simulation of five real-world head impact events in rugby, to assess the level of protection provided by a novel foam headguard, the N-Pro. The University College Dublin Brain Trauma Model (UCDBTM) was used to estimate the peak resultant head accelerations and brain tissue responses in different head impact scenarios. The input kinematics were obtained from two sources: video analysis of impact events, and real-time data obtained through instrumented mouthguards. The impact events were simulated under both unprotected and protected conditions. All simulations were performed against a rigid, non-compliant surface model. The results obtained in this study demonstrate the significant potential of the N-Pro in reducing peak head accelerations and brain tissue stress/strain responses by up to c. 70% compared to unprotected head impacts. This study highlights the headguard’s promising potential to reduce the severity of impact-related injuries by effectively attenuating stresses and strains, as well as linear and rotational kinematics. Additionally, the study supports the recommendation in the literature that kinematic data collected from wearable sensors should be supplemented by video analysis to improve accident reconstructions.

## 1. Introduction

In recent years, the potential long-term effects of concussions and repeated sub-concussive head impacts have sparked growing concerns within the athletic community. Numerous athletes have shared the complications they have faced from long exposure to high impact collisions, resulting in the diagnosis of early onset dementia and other neurological disorders [1,2,3]. Chronic traumatic encephalopathy (CTE), a condition resulting from repeated concussions and characterised by symptoms such as depression, memory loss, concentration problems, personality disorders, and other cognitive impairments, has been identified as a particular worry [4,5]. This heightened awareness has led researchers to investigate the health consequences of concussions in athletes, and to explore whether soft-shell head protection can help to prevent them or reduce the severity of such injuries.

A concussion is a subset of diffuse traumatic brain injury (TBI) that occurs when the brain absorbs sufficient kinetic energy following a direct or indirect impact, so it undergoes significant motion and intracranial stresses and strains within the skull. Consequently, a temporary disturbance of brain function occurs [5,6]. Early research suggested that concussive brain injury was caused primarily by linear acceleration forces, resulting in a back-and-forth motion of the brain within the skull [7]. However, experimental studies identified that rotational kinematics are correlated with a greater severity of TBI [8,9,10]. This is explained by the shearing and stretching of the brain tissue that rotational forces can cause, resulting in high levels of strain within the brain, and subsequent injury [11,12]. Additionally, Ommaya [13], four decades ago, explained that rotational forces can produce both focal and diffuse brain injuries, while translational force is limited to focal effects. Injury statistics have found the most common accident situations involve an oblique impact, which gives rise to both linear and rotational head kinematics [10]. These types of impacts are observed frequently in contact sports during tackles or falls, where a person’s head is subjected to both linear and rotational accelerations, resulting in the risk of a sport related concussion (SRC).

Sport Related Concussions (SRCs) are a more complicated type of concussion as athletes can be subjected to sub-concussive head impacts on a regular basis. This is distinguishable from a single-impact TBI, as repeated impacts may contribute to cumulative brain trauma that eventually manifests as a long-term impairment [14]. Moreover, repeated concussive and sub-concussive head injuries may cause a range of neurological sequelae, including post-traumatic stress disorder (PTSD), chronic traumatic encephalopathy, post-concussion syndrome, and dementia [14,15]. The physical nature of the sport, combined with the size and strength of players in contact and collision team sports such as rugby, pose a significant risk of concussive injuries. According to the 2020–2021 English Professional Brain Injury Surveillance Project, concussions were the most often reported match injury for the tenth consecutive season, accounting for 28% of all match injuries and having an incidence rate of 22.2 concussions per 1000 h [16]. A similar study from Ireland found that concussions occurred during matches at a rate of 20 per 1000 player-match hours [17]. The risk of injury is particularly high in the tackle, scrum, and ruck phases of the game [18,19]. Specifically, research suggests that 50–70% of all concussions are associated with tackles [16,17,18,19,20,21]. These findings, and the heightened awareness of the risks that come with TBIs, have led researchers to further investigate the consequences of concussions in athletes, and to explore whether soft-shell head protection can help to prevent them.

Earlier studies of concussion in rugby have led to the efficacy of traditional headguard models being called into question [3,22]. A study conducted by Knouse et al. evaluated the effectiveness of branded rugby headgear in attenuating repetitive linear impact forces [4]. They compared the attenuation capabilities of two common types of headgear, Honeycomb and Vanguard, using an unprotected headform as control. Diverse International Rugby Board (IRB) approved headgears of these two types were dropped from a fixed height of 30 cm, in accordance with the International Rugby Board (IRB) test standards, to measure force attenuation at two different impact sites (posterior and parietal-lateral regions). The test involved measuring the headform’s peak instantaneous acceleration and Gadd Severity Index (GSI) on contacting an MEP pad. Compared to the unprotected headform, both types of headgear attenuated impact forces. However, after ten drops, the ability to absorb repeated linear impacts decreased by approximately 10 to 36%, depending on the type of headguard and the impact location, and a decrease in performance was evident after just one impact. This and other similar studies [22,23,24,25,26] identified the need to research different materials and headgear designs that would successfully attenuate repetitive impact forces experienced during contact sports without losing effectiveness over time. McIntosh et al. [21] suggested that the “memory effect” of soft, closed-cell foam used in rugby gear may prevent the material from regaining its original thickness after impact, leading to a reduction in headgear effectiveness over time. Therefore, recent research has evaluated several materials for their effectiveness in improving impact absorption, as well as leading to the development of innovative headgear designs that can combat the current limitations.

Draper et al. [24] conducted a study to evaluate the performance of six commercially available headguards from five different commercial manufacturers, including two size variations (medium and large) for one brand. The headguards assessed were the CCC Ventilator, Kukri, 2nd Skull, N-Pro, and both medium and large version of Gamebreaker Pro. The primary objective of their study was to assess the ability of the different headguards to attenuate peak linear accelerations (PLA) and the head injury criterion (HIC) scores were used to evaluate their relative effectiveness. Those results indicated that the N-Pro and the Gamebreaker Pro headguards significantly reduced both PLA and HIC than did the other three headguard designs. They also indicated that the Gamebreaker Pro designs offered even better reductions in PLA and HIC than the N-Pro design: this was attributed to the thicker layers of protection in the Gamebreaker Pro. In addition, Stitt et al. [25] conducted a study to examine the potential of headguards to mitigate both linear and rotational accelerations. Their experimental investigation involved analysing peak accelerations that occurred when headguards were attached to instrumented headforms and dropped vertically from four different heights, at various angles, and on different contact points on the head. Five commercially available headguards were tested in that study: CCC Reinforced, Gilber Falcon 200, 2nd Skull, N-Pro and Gamebreaker Pro. The results demonstrated clearly that both the N-Pro and the Gamebreaker Pro significantly reduced the peak linear accelerations (PLA) and peak rotational accelerations (PRA) than did either of the other three headguard designs. In light of those previous studies, the present work does not attempt to compare the performance of alternative commercially available headguards. Nonetheless, some general observations can be made in respect of alternative soft-shell headguard designs. Most tend to rely on using various viscoelastic foam pads that are incorporated into a laced-up form that provide close contact with a wearer’s head. Some of the designs that are certified for use by World Rugby provide greater levels of coverage to vulnerable regions of the head than others. A thorough comprehensive analysis of the relative efficacy of each headguard design and identifying particular aspects of each design that might merit improvement is beyond the scope of this present study.

This present study was completed in conjunction with a leading headguard manufacturer, N-Pro, and focuses on their headguard as a representative design of advanced head protection worn in rugby. This headguard differs from closed-cell foam headguards due to its proprietary viscoelastic polymer foam, Defentex, that is incorporated into the padding [26]. The material was designed specifically to help attenuate head impacts and aid in the mitigation of concussive injuries. The incorporation of Defentex allows the absorption of impact energy even without a hard outer shell. The headguard is made up of soft, elastic segments that are capable of absorbing energy, and stiffer segments that provide durability and stability [26]. When compared with two other commercial headguards, the N-Pro significantly reduced linear and rotational accelerations of the head following impact [24,25,26]. Results from mechanical drop tests have shown the headguard’s ability to reduce linear accelerations experienced by the head during an impact by up to 75%, compared to other commercially available headguards, and rotational accelerations by an average of 34% compared to a bare headform [26].

As discussed above for SRCs, athletes may be exposed to repeated head impacts of a sub-concussive nature on a regular basis. Recent discussion regarding diagnostic criteria for mild traumatic brain injury (mTBI) caused by repetitive concussive and sub-concussive head impacts highlights the heterogeneity of the signs and symptoms that exist for SRC [15,27]. Advancements in side-line and clinical assessment of SRC in rugby include the 6th iteration of the Sports Concussion Assessment Tool (SCAT-6) following the 2022 consensus meeting of the Concussion in Sport Group [28]. While this offers improvements to the evaluation of athletes following a potential SRC, there remains a need to develop enhanced diagnostic tests and objective biological markers of SRC that overcome current drawbacks in outcome sensitivity and subjectivity. Ongoing research into the neurological effects of repeated head impact exposures in athletes has leveraged neuroimaging, neurological blood biomarkers, and oculomotor performance [27,28,29,30,31] amid efforts to determine whether a threshold for adverse events exists for exposure to sub-concussive impacts. Laboratory testing and previous in-silico examinations of SRC have proposed tolerances for brain injury based on the kinematic and tissue responses of the head and brain under loading [8,32,33,34,35,36,37]. Real-time monitoring of head impact exposure alongside enhanced clinical insights into the brain’s structural and metabolic response to repetitive sub-concussive loading would provide further clarity into potential upper and lower thresholds above or below which SRCs may or may not occur for a specific population. This is discussed in further detail following our predictions for real-world injury events in rugby, alongside recommendations on how real-world clinical evidence can be leveraged in conjunction with finite element (FE) predictions of brain deformation to offer improvements for prevention strategies against SRC.

Although mechanical impact tests are useful to determine whether a headguard attenuates force, they do not consider the stresses and strains experienced by the brain during an impact. As mentioned previously, TBI is not only characterized by movement of the brain relative to the skull following an impact, but also by high strains following the deformation of brain tissue [11]. Therefore, headguards must also be tested for their effect on brain tissue response. FE models have been instrumental in the study and understanding of head injury biomechanics [36,37,38], as well as the protective capabilities of helmets used in sports [38,39,40,41,42,43,44,45], such as ice hockey, cycling, motorcycling, skiing and equestrian sports. The models are generally developed from slices of computed tomography (CT) scans or magnetic resonance imaging (MRI) images of human subjects [38,39]. Furthermore, their accuracy has been validated by performing comparative tests with results from cadaveric experiments, and the models are continuously being improved to better represent the geometry, anatomy and components of the brain [40]. These advances make them powerful tools for studying head impact biomechanics.

The UCDBTM is a finite element model developed by Horgan & Gilchrist [38] to study head impact biomechanics, and has been used to evaluate the efficacy of headgear for rugby [43], to establish the performance of equestrian helmets [37,44], estimate the brain tissue response while a soccer player heads the ball, and to compare different helmet designs for ice hockey goal keeper helmets [41]. Other similar three-dimensional computational models exist and these include NHTSA’s SIMON (Simulated Injury Monitor) model [46], which has been used extensively to simulate head protection during car crash events. Duckworth et al. [47] created a three-dimensional finite element model of a rodent brain to evaluate cerebral vascular injury and to predict microbleed locations. Zhou et al. [48], Zhang et al. [49] and Gadjari et al. [50] also created very refined finite element models of the human brain to properly represent the cerebral ventricles, sulci and gyri, and to account more accurately for the fluid behaviour of the cerebrospinal fluid (CSF).

This study proposes that simulating head impact events in rugby, under both unprotected and protected loading conditions, will advance our understanding of the levels of protection provided by energy-absorbing foam headgear. Such knowledge will lead to improvements in the design of headguards used in rugby, ultimately benefiting player welfare.

## 2. Materials and Methods

### 2.1. Kinematic Impact Data

The kinematic data were collected using instrumented mouthguards from Prevent Biometrics, as well as high quality video footage. The Prevent Biometrics data were analysed separately for two cases, and combined with video footage for two cases, while video footage alone was used for one case. Kinovea software was used to extract kinematic data from the recorded video footage, for subsequent analysis.

#### 2.1.1. Video Analysis

The head kinematic data used in this study come from two different sources: video analysis of real-world head impacts in rugby as seen in publicly available footage, and from the use of instrumented mouthguards. The video data used in this study were obtained from freely available broadcast match data. The three specific cases selected for video analysis were chosen based on their compatibility with analysis using Kinovea, ensuring a clear and visible area of impact and minimal camera movement. To avoid inaccuracies in accident reconstruction, the impact cases were selected carefully from several videos based on specific criteria: minimal camera motion, a clear unobstructed view of a direct impact between the head and ground, and a clearly visible head impact location. Four additional cases that were available with video footage and mouthguard data. However, the impact event in those cases were variously obstructed, blurry or affected by excessive camera movement. Several studies have previously assessed the validity of Kinovea for biomechanical analysis. One study [51] reported a variance of less than 10% compared to an established motion capture system, while several other studies [52,53,54] have found it to be an effective tool for evaluating athletic performance. Based on these criteria, one head impact case based solely on video was selected along with two cases where both video footage and instrumented mouthguard data were available. The impact details for each case are summarised in Table 1. To determine the kinematic values associated with impact, analysis of the available video footage was performed using Kinovea 0.9.5. This software was specifically designed for sports analysis and has been used by various researchers to perform movement analysis [41,42,53].

**Figure 1:**
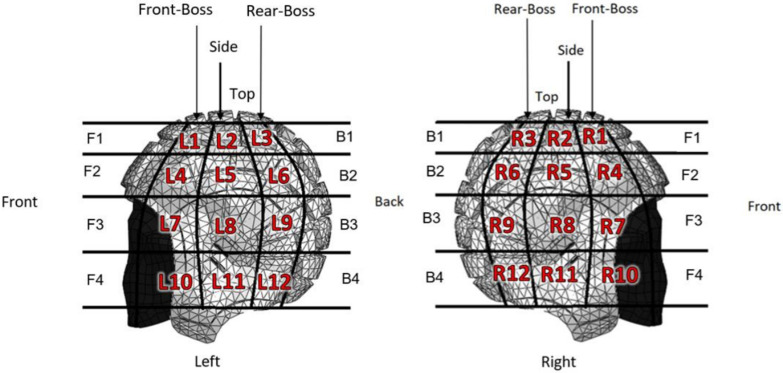
Left and right view of the segmented headguard used as a reference grid to determine impact locations for accident reconstruction (Reprinted from Ref. [42]).

**Figure 2:**
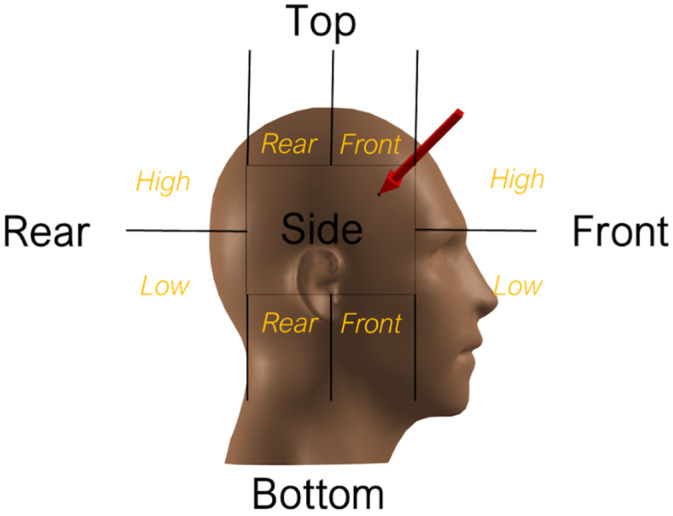
Right view of the segmented head used as a reference grid to determine impact locations for accident reconstruction for instrumented mouthguard (image provided by Prevent Biometrics).

Impact location was determined using a reference grid as illustrated in Figure 1 [41,42,43] and Figure 2. For each of the three real-world impact cases, distance measurements were converted from pixels to meters using known values, and horizontal and vertical distance measurements were calibrated to determine displacements and velocities. Players’ known heights, and pitch markings in the video footage, were used as scaling references in the video analysis.

#### 2.1.2. Instrumented Mouthguard

This study also used four sets of data, as summarised in Table 2, which were obtained using Prevent Biometrics’ Impact Monitoring Mouthguard (IMM) to explore an alternative method for obtaining kinematic data. Two of these sets (Cases 2 and 3) were the same as Cases 2 and 3 noted in Table 1 (i.e., video cases), while the two other cases (Cases 4 and 5) did not include the use of video data. The Prevent Biometrics IMM estimates the time-varying accelerations and velocities of head movement due to an array of sensors that are embedded in the mouthguard, and these are typically used in contact sports and military applications [55,56,57,58,59]. Studies evaluating the accuracy of the instrumented mouthguards that were used in this present study essentially concluded that the kinematic data are estimated with high accuracy. One such study [60] showed that the mouthguard from Prevent Biometrics tended to underpredict kinematic data when compared to a reference sensor, although a competitor’s mouthguard (Diversified Technical Systems, DTS) tended to overpredict the data. The Prevent Biometrics mouthguard agreed more closely with a reference sensor. A separate comparative study [61] to evaluate mouthguards from four separate manufacturers concluded that the mouthguard from Prevent Biometrics had the highest feasibility ratings. Moreover, the rigid coupling between a person’s upper teeth and their skull has been shown [62] to be a viable way to measure head impact kinematics in order to assist with evaluating the biomechanics of concussion.

**Figure 3:**
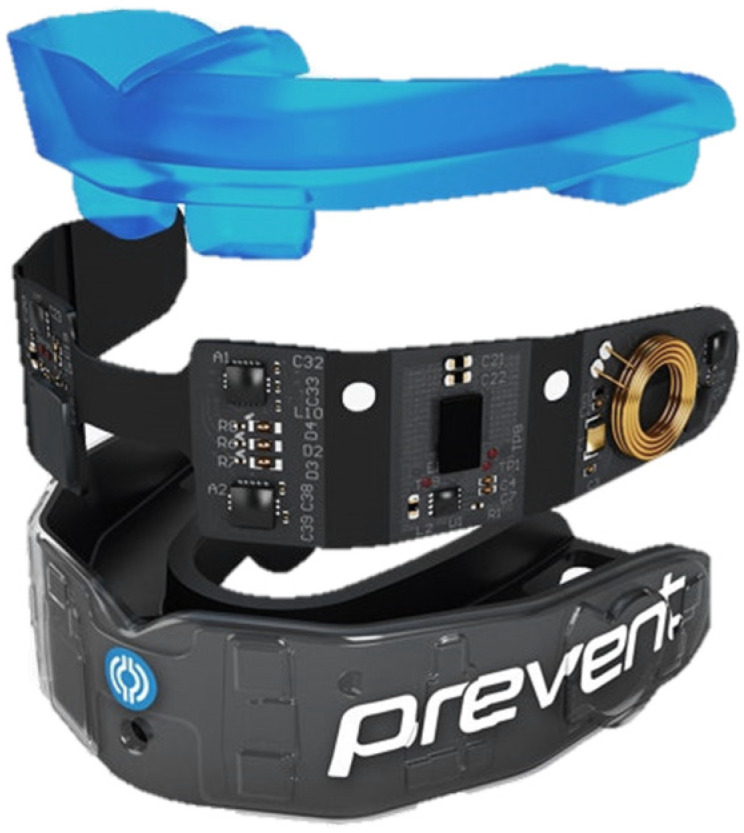
Impact Monitoring Mouthguard (IMM, Prevent Biometrics) Reprinted from Ref. [63].

Sensors embedded in the IMM, as seen in Figure 3, estimate the time-varying kinematics experienced by players’ heads during training and match-time, providing real-time head impact or inertial kinematic data. Having been tested for reliability and validity, Prevent Biometrics’ IMM is increasingly being used for head impact monitoring [63]. The measurable outputs of interest used in this study were the location and direction of impact, as well as linear and angular acceleration time histories as summarised in Table 2. Data were collected and stored on local memory when a buffering accelerometer measurement exceeded 8 g on a single axis. The system recorded data at a sampling rate of 3200 Hz and used standard textbook rigid body transforms to translate three axes each of measured linear acceleration and angular velocity from a player’s mouth to the full six degree of freedom estimates for time-varying head CoM kinematics over a 50 msec duration [63].

As explained further below in Section 3, both Cases 4 and 5 have no video footage to accompany the Prevent IMM data; therefore, the cases were simulated using the impact locations as specified by Prevent IMM in their metadata, as indicated in Table 2.

### 2.2. Finite Element Modelling

**Figure 4:**
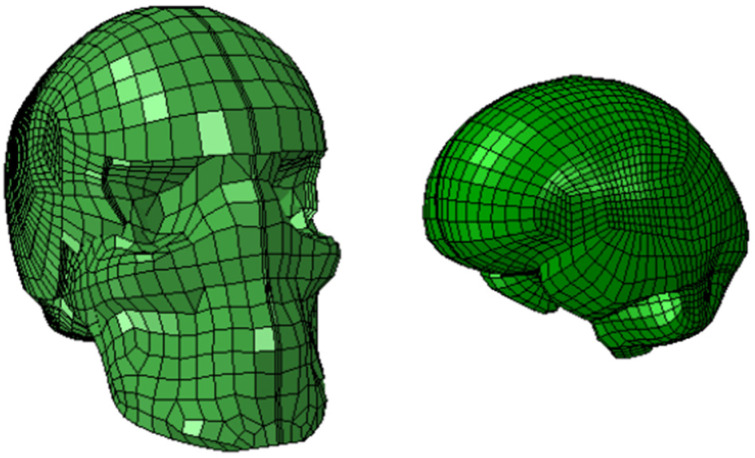
The head (**left**) and brain (**right**) of the UCDBTM.

The UCDBTM was used in this study to model the human head and brain. The FE head model, shown in Figure 4, was developed by Horgan and Gilchrist to study head impact biomechanics [38,64]. To generate an accurate representation of the human head and brain, the geometry of the model was created based on CT scans and MRI scans of male human cadavers. The model consists of approximately 28,000 elements, and the elements are both shell and solid elements, all of which are hexahedral, and it incorporates several key components of the brain, including the skull, scalp, falx, cerebrospinal fluid (CSF), pia mater, tentorium cerebelli, cerebellum, gray matter, and white matter. These components provide a comprehensive representation of the brain and its various features. To ensure the accuracy of the model, following a mesh sensitivity analysis, it was validated through the simulation of multiple head drop experiments performed on a commercial headform, details of which are provided elsewhere [38,64] and, for the headguard, in Section 2.3 below. The model’s predictions were also compared to the experimental pressure time-histories obtained by Nahum et al. [65], Hardy et al. [66] and Trosseille et al. [67] and the results from CT scans for the real-world accidents. Additionally, only the N-Pro headguard was examined in this present study in the wider context of a World Rugby Law 4 Trial [68]. Direct comparisons of the N-Pro headguard against other commercially available headguards have already been studied as noted above [23,24,25,26] and is beyond the scope of this present study.

The material properties of the UCDBTM are shown in Table 3 and Table 4 [38,64]. The brain tissue was modelled using a linearly viscoelastic model combined with large deformation theory to account for brain material being highly damped. The behaviour of this material was characterised as viscoelastic in shear with a deviatoric stress rate dependent on the shear relaxation modulus [36]. The compressive behaviour of the brain tissue was considered to be elastic. The CSF layer was modelled using solid elements with a low shear modulus.

**Figure 5:**
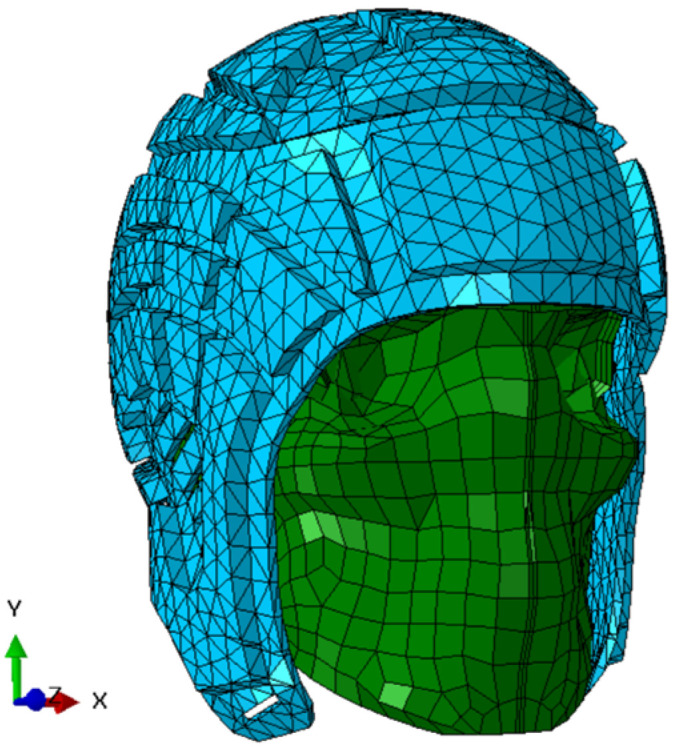
The UCDBTM fitted with a FE model of the N-Pro headguard.

One specific headguard design was considered in this present investigation, namely the N-Pro headguard. A finite element model of this was created and integrated with the UCDBTM, as shown in Figure 5, while the constitutive properties used in the finite element model of the N-Pro headguard are included in Table 3.

**Figure 6:**
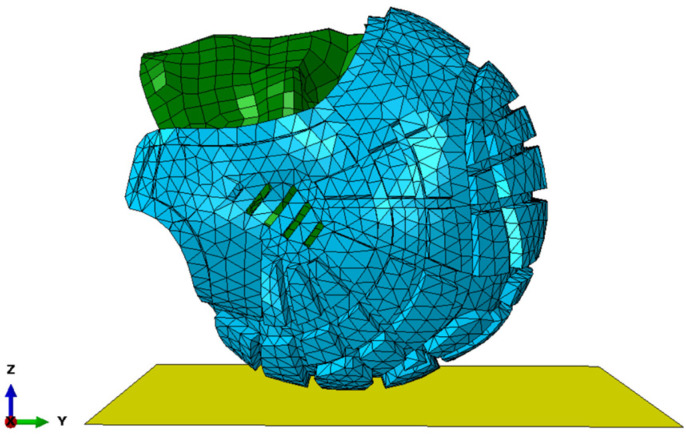
Rear contact of UCDBTM (blue) with the rigid impact surface (yellow).

The head impact events were all simulated using a non-compliant rigid impact surface. The rationale for this is that it is consistent with World Rugby regulations, which specify a rigid impact surface must be used for impact attenuation testing of headgear [69]. The rigid impact surface was simply created by drawing a 2-dimensional, rigid, undeformed, region that was sufficiently large for the head to contact, as illustrated in Figure 6.

### 2.3. Headguard Model Validation

The headguard finite element model was validated against previously reported experiments of an instrumented headform (mass = 4.7 kg) fitted with the headguard that was dropped vertically from a height of 0.3 m under gravity in order to induce linear impacts to the crown, forehead (left and right), and temple (left and right) regions [26]. The maximum linear accelerations were extracted from the centre of mass of the headform for each impact location. These experiments were recreated in Abaqus using the UCDBTM fitted with the headguard finite element model. Impacts from 0.3 m drop heights under gravity were simulated by applying a predefined velocity of 2.43 m/s at the moment of impact. Due to the symmetry of the model, only the right temple and left forehead were simulated. The maximum linear acceleration from the UCDBTM’s centre of mass was extracted and compared against the experimentally measured accelerations [26]. In the absence of explicit manufacturer’s data on the dynamic mechanical properties of the headguard material, an inverse optimisation approach was taken using parametric simulations to infer an equivalent Young’s modulus and Poisson’s ratio such that an optimal response was achieved for all of the peak linear acceleration at the UCDBTM’s centre of mass (CoM) for the crown, temple and forehead impact locations. Best agreement with experiment was found for an elastic modulus of 5 MPa, which is consistent with independently measured elastic modulus values for similar low density porous foams tested under large strain dynamic compression [70].

**Figure 7:**
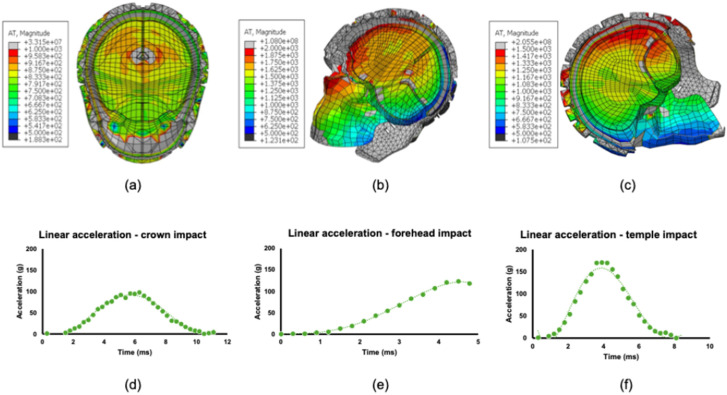
Contour maps of cross sections of the UCDBTM showing linear accelerations at the time of peak linear acceleration for (**a**) crown, (**b**) forehead, and (**c**) temple impacts at 2.43 m/s with a rigid surface. The acceleration-time (g-ms) profiles for (**d**) crown, (**e**) forehead, and (**f**) temple impacts are also shown.

Figure 7 shows the instant of peak linear acceleration (PLA) for all three impacts, i.e., against crown, forehead and temple regions. As can be seen from Figure 7a,d in the case of the crown impact, most sections of the brain show a linear acceleration of approximately 800 m/s^2^, or about 82 g. The peak linear acceleration taken from the elements around the CoM is 95 g as compared to the experimental results of 140 g (c.f. Table 5). The highest acceleration is shown by the elements closest to the face. The forehead impact (Figure 7b,e) shows that most areas of the brain experience a linear acceleration of about 120 g, with PLA around the CoM experiencing approximately 135 g. The maximum acceleration is shown by the elements closest to the point of impact in the frontal lobe. For the temple impact (Figure 7c,f), the largest sections of the brain have linear accelerations of approximately 150 g, with the elements around the CoM experiencing a PLA of 162 g. This is the highest PLA out of all three instances, unlike the experimental results [23] which showed the highest values of PLA for the crown impact.

### 2.4. Accident Reconstruction and Modelling

The UCDBTM was used to simulate five real-world head impacts using Abaqus CAE 2023. Each impact position was established by orienting the head model to match that of each accident. All impact positions for the five protected and five unprotected cases are shown in Table A1 (Appendix). The UCDBTM coordinate system (origin at the CoM of the model) was used as the axes of rotation for the rotational velocities, with the *x*-axis pointing posterior to anterior, *y*-axis pointing left to right and *z*-axis pointing down. The recordings from the Prevent IMM used the SAEJ211 coordinate system and were rotated to the same UCDBTM coordinate system for ease of comparison. The global coordinate system in Abaqus was used for the linear velocities as the impact surface remains in the same location with respect to it, regardless of the head model orientation. The linear and angular velocities were used as velocity predefined field inputs (Table 6), and appropriate interactions and constraints between surfaces and model components were established to recreate the accidents as accurately as possible.

To prevent the lack of neck restraint from affecting the simulation results, simulations ran for a short duration (up to 10–20 ms) and were stopped once the model rebounded following impact. All five of the impact cases that were simulated were recreated using an unprotected head (UCDBTM) and protected head (UCDBTM fitted with the N-Pro headguard FE model) against a rigid non-compliant impact surface. The UCDBTM was used to calculate the peak resultant head accelerations: peak linear acceleration (PLA) as recorded by the accelerometers fixed at the head’s CoM; as well as the peak brain tissue responses: von Mise’s stress (VMS) and maximum principal strain (MPS) by isolating the elements of the grey, white, and ventricular matter, cerebellum, brain stem and cerebral hemispheres.

## 3. Results

The results of the head impact video reconstructions (Cases 1–3) and from Prevent IMM (Cases 2–5) are presented and summarised below. The tables in this section outline the linear and rotational impact velocities used as inputs for the simulations, as well as the maximum resultant values as predicted by Abaqus CAE 2023, for the parameters of interest (PLA, MPS and VMS).

### 3.1. Case 1

**Figure 8:**
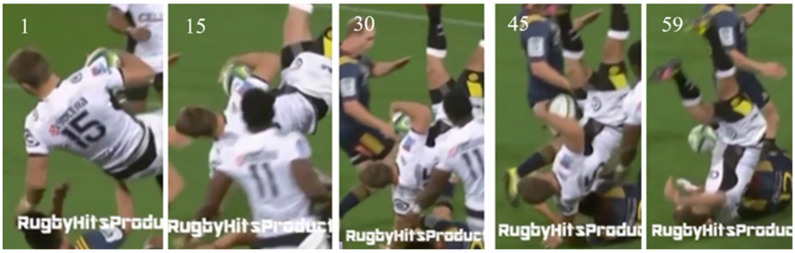
Sequential images of head impact from Case 1 over a period of 2.37 s. The frame rate of the video was 25 frames per second. Each image is identified by its frame number at the top left corner. The last image shows the player impacting the ground at region L3 of the reference grid (Figure 1).

Case 1 was based on a head to ground collision as seen in publicly available footage of a 2016 Highlanders vs. Sharks rugby match. Table 1 and Table 7 summarises the event details and provides a link to the corresponding video footage. Based on the reference grid shown in Figure 1 and the sequential images displayed in Figure 8, the impact site was identified as the back of the head in the L3 region. The figures in Table A1 (Appendix) illustrate how the UCDBTM with and without the headguard FE model was rotated and translated into the correct position for accident reconstruction. The figures in Table A2 and Table A3 (Appendix), and the quantitative results shown in Table 8 illustrate how the brain tissue deformed significantly less (i.e., lower levels of strain and stress) than in the unprotected scenario, especially along the longitudinal fissure and the left parietal and frontal lobes.

### 3.2. Case 2

**Figure 9:**
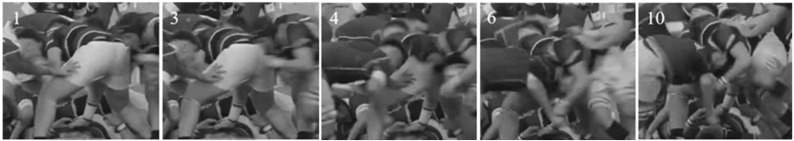
Sequential images of head impact from Case 2 over a period of 1.10 s. The frame rate of the video was 30 frames per second. Each image is identified by its frame number at the top left corner. The last image shows the player impacting the ground at region Top Rear of the reference grid (Figure 2).

Case 2 was based on a head to shoulder collision as seen in publicly available footage of a schools’ rugby match. Table 1 and Table 9 summarise the event details and provides a link to the corresponding video. Based on the reference grid shown in Figure 2 and the sequential images displayed in Figure 9, the impact site was identified as high up on the back on the head in the Top Rear region. The figures in Table A1 (Appendix) illustrate how the UCDBTM with and without the headguard FE model was rotated and translated into the correct position for accident reconstruction. The stress and strain distributions over the brain were notably reduced, particularly in the temporal region and brain stem, as demonstrated by the corresponding figures in Table A2 and Table A3 (Appendix) and as summarised in Table 10.

### 3.3. Case 3

**Figure 10:**
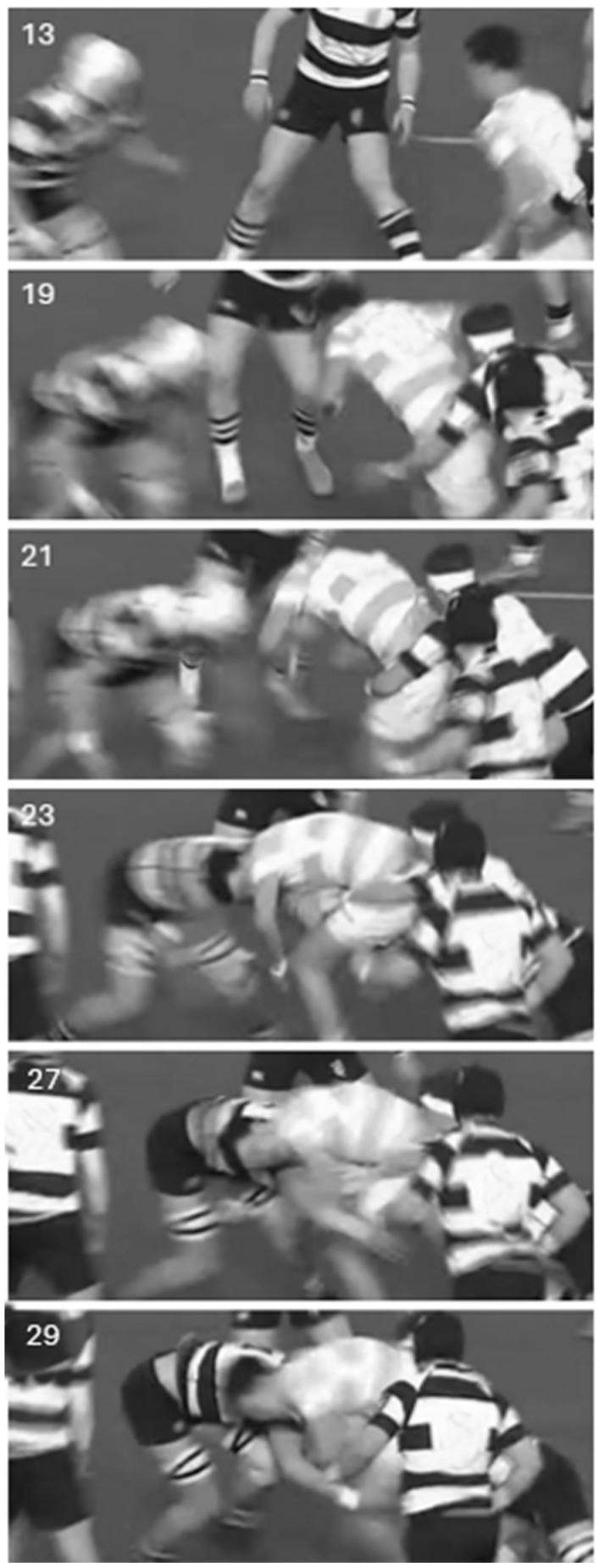
Sequential images of head impact from Case 3 over a period of 0.53 s. The frame rate of the video was 30 frames per second. Each image is identified by its frame number at the top left corner. The last image shows the player impacting the floor at region Top Rear of the reference grid (Figure 2).

Case 3 was based on a head to shoulder collision as seen in publicly available footage of a schools’ rugby match. Table 1 and Table 11 summarise the event details and provides a link to the corresponding video footage. Based on the reference grid shown in Figure 2 and the sequential images displayed in Figure 10, the impact site was identified as high back on the head in the Top Rear region. The figures in Table A1 (Appendix) illustrate how the UCDBTM with and without the headguard FE model was rotated and translated into the correct position for accident reconstruction. The figures in Table A2 and Table A3 (Appendix) corresponding to Case 3, illustrate the attenuation of both stress and strain distributions in the right hemisphere, particularly in the parietal lobe using the Prevent IMM data. Using the collected Kinovea data, a reduction in both stress and strain distributions in the brain stem were also predicted, as illustrated in Table A2 and Table A3 (Appendix). Table 12 summarises the stresses, strains and accelerations within the unprotected head during the Case 3 impact, along with the changes (i.e., reductions) associated with wearing the N-Pro headguard.

### 3.4. Case 4

Case 4 was based on a head impact recorded by an instrumented mouthguard. No video footage was available for this impact. Therefore, the impact site was region Front High in the reference grid (Figure 2) based on the data provided by the IMM system, as summarised in Table 2 and Table 13. The figures in Table A1 (Appendix) illustrate how the UCDBTM with and without the headguard FE model was rotated and translated into the correct position for accident reconstruction. Furthermore, as seen in Table A2 and Table A3 (Appendix) and summarised in Table 14, the headguard significantly reduced the stress and strain concentrations at the impact site, particularly in the right parietal lobe.

### 3.5. Case 5

Similar to Case 4, Case 5 was based on a head impact recorded by a Prevent Biometrics’ instrumented mouthguard. No video footage was available for this impact. Therefore, the impact site was region Left High in the reference grid (Figure 2) based on the data provided by the IMM system, as summarised in Table 2, Table 15 and Table 16. The figures in Table A1 (Appendix) illustrate how the UCDBTM with and without the headguard FE model was rotated and translated into the correct position for accident reconstruction. Furthermore, as seen in Table A2 and Table A3 (Appendix), the headguard significantly reduced the stress and strain concentration and the impact site, particularly in the right parietal lobe.

**Figure 11:**
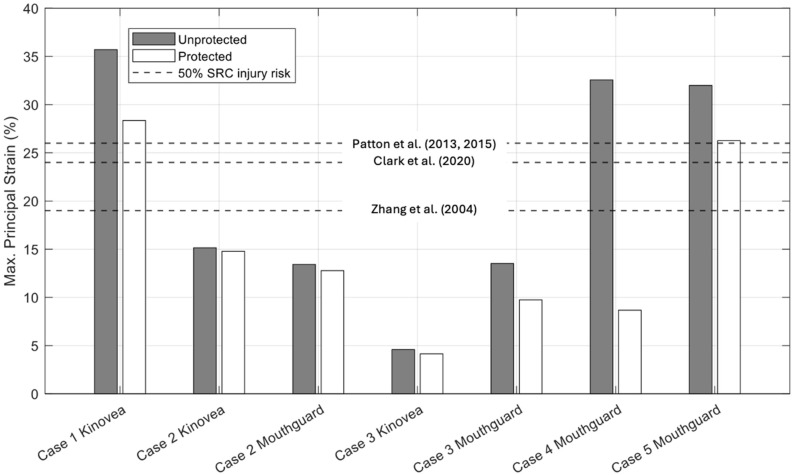
Results of peak Max. Principal Strain values for each of the four real-world impact cases. The three black dashed lines indicate various threshold values for 50% SRC injury risk (Zhang et al. 2004 [9], Patton et al., 2013, 2015 [35,36], Clark et al. (2020) [37]). Note that Cases 2 and 3 were reconstructed using both video and mouthguard sensor data, while Cases 1, 4 and 5 only relied on a single set of kinematic data, i.e., either video or mouthguard data.

**Figure 12:**
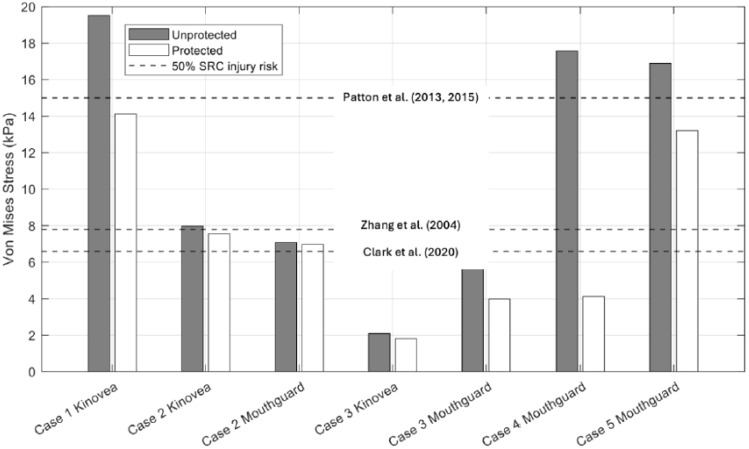
Results of mean Von Mise’s stress values for each of the four real-world impact cases on a rigid surface. The three black dashed lines indicate various threshold values for 50% SRC injury risk (Zhang et al. (2004) [9], Patton et al. (2013, 2015) [35,36], Clark et al. (2020) [37]). Note that Cases 2 and 3 were reconstructed using both video and mouthguard sensor data, while Cases 1, 4 and 5 only relied on a single set of kinematic data.

**Figure 13:**
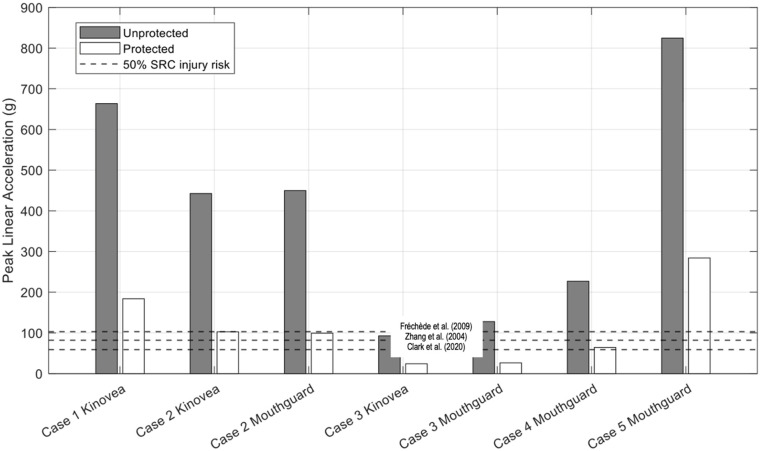
Results of mean Peak Linear Acceleration values for each of the four real-world impact cases on a rigid surface. The three black dashed lines indicate various threshold values for 50% SRC injury risk (Zhang et al. [9], Fréchède et al. [34], Clark et al. [37]). Note that Cases 2 and 3 were reconstructed using both video and mouthguard sensor data, while Cases 1, 4 and 5 only relied on a single set of kinematic data.

Figure 11, Figure 12 and Figure 13 below illustrate the peak results predicted by Abaqus (PLA, VMS, MPS) for protected and unprotected simulations of each event.

## 4. Discussion

### 4.1. Case 1

Based on the results from Table 8 and Figure 11, Figure 12 and Figure 13, Case 1 has the highest MPS, VMS and PLA in the unprotected and protected cases of all five cases, with results of 35.70%, 19.52 kPa and 663.66 g, respectively, for the unprotected impact. It is evident that the N-Pro headguard considerably reduced the peak VMS, MPS, and PLA, as well as the severity of the impact. By fitting the headguard to the UCDBTM, the PLA decreased by 72.31% from 663.66 g to 183.78 g. Both VMS and MPS showed similar reductions (27.66% and 20.59%, respectively). The reduction in brain kinematics is consistent with experimental mechanical impact tests reported separately by Ganly et al. [26]. Based on the obtained results and the proposed brain tissue response tolerances [9,35,36,37], an individual not using the headguard faced a higher risk of sustaining a head injury in this case. Nonetheless, even if they had been wearing the headguard, they were at risk of receiving a concussive head injury, albeit less severe.

### 4.2. Case 2

Data collected by Prevent IMM and Kinovea revealed that Case 2 predicted the smallest reductions in MPS and VMS irrespective of whether mouthguard or video data were used to quantify the impact conditions, as shown in Table 10 and Figure 11, Figure 12 and Figure 13. For the unprotected condition using Prevent IMM, the recorded MPS, VMS, and PLA values were 13.42%, 7.09 kPa, and 499.86 g, respectively. Relatively small differences were observed between the Prevent IMM and Kinovea data for both protected and unprotected cases. Consequently, the differences in predictions of MPS and VMS between the two data collection methods were also small, while the unprotected simulations consistently predicted higher values than the corresponding protected simulations. The use of the N-Pro headguard led to a substantial reduction in PLA, with decreases of 77.91% and 76.81% predicted when using the Kinovea and Prevent IMM kinematic data, respectively.

### 4.3. Case 3

Case 3 exhibited the least severe impact conditions of all five cases. This is clearly due to the slower impact velocities, particularly the resultant linear velocities of 2.316 m/s and 0.87 m/s observed in the Prevent IMM data and Kinovea data, respectively (see Table 11). Notably, the Kinovea data demonstrated significantly smaller values along the *y*-axis (or rightward direction) compared to the Prevent IMM data. A significant reduction in PLA was observed using both data sources, namely in excess of 70%, as illustrated in Table 12 and Figure 13. In the protected simulations, the Prevent IMM data showed reductions in MPS and VMS of 27.96% and 41.32%, respectively. However, due to the substantially lower initial velocities in the Kinovea data, the reductions in MPS and VMS were comparatively smaller, likely reflecting the already lower baseline values. Importantly, all results from Case 3 were significantly below the SRC injury thresholds [9,35,36,37].

### 4.4. Case 4

Case 4 demonstrated the highest reductions in both MPS and VMS when wearing the headguard, at 73.36% and 76.51%, respectively, along with a significant attenuation of PLA by 71.65%, likely due to the extensive padding over the temples. Specifically, MPS decreased from 32.56% to 8.67%, and VMS decreased from 17.58 kPa to 4.13 kPa, as shown in Table 14 and Figure 11, Figure 12 and Figure 13. The use of the headguard further resulted in a 71.65% reduction in PLA. Notably, in the unprotected scenario, all MPS, VMS and PLA levels exceeded the 50% risk thresholds for SRC injury as reported in prior studies [9,35,36,37]. However, with the inclusion of the headguard, the corresponding MPS, VMS and PLA values all fell below the 50% injury risk thresholds [9,35,36,37].

### 4.5. Case 5

Case 5 has the highest rotational velocity of all five cases, with a rotational velocity of 29.77 rad/s. As shown from the results in Table 16 and Figure 11, Figure 12 and Figure 13, the percentage reduction in MPS and VMS due to the inclusion of the N-Pro headguard are both significant, reducing by 40.96% and 21.83%, respectively. Additionally, with the inclusion of the N-Pro headguard, PLA is reduced by 65.54%. However, when comparing the peak brain deformation properties to proposed thresholds for SRC injury risk, all three predictors exceed the 50% injury thresholds while either wearing or not wearing the headguard.

### 4.6. Thresholds for Concussion

The FE predictions for each of the injury cases examined above highlight the reductions in peak resultant head accelerations (PLA) and peak brain tissue responses (MPA, VMS) when an energy-absorbing designed headguard, such as N-Pro, is included in impact simulations. Various researchers have suggested kinematic thresholds for SRC in rugby and other contact sports using either peak linear or angular acceleration metrics generated from laboratory reconstructions of real-world injury events, as summarised in Table 17. Similarly, several studies which have examined the brain tissue mechanical responses due to injurious kinematic loading have recommended stress and strain based tolerances for the occurrence of SRC injuries. With particular regard to research involving rugby, 27 cases of concussive head impact events in Australian Rules Football and rugby were reconstructed following video analysis to estimate the associated linear and rotational head accelerations of each event [34]. Classification of an SRC for each case was based on the causes of impact, the subsequent symptoms and, as per the 2004 definition from the Concussion in Sport group meeting [71], recently revised at the 2022 consensus meeting [26]. The reconstructions of the same 27 cases as described in [34] were subsequently examined further [35,36] using computational techniques, alongside 13 non-injurious cases, to assess the effectiveness of several brain tissue response metrics in predicting SRC.

Laboratory reconstruction of head impact events based on video review and analysis allows for impact kinematics to be estimated for a particular event. However, improved accuracy in measured kinematics can be achieved using real-time instrumented mouthguards [72]. Instrumented mouthguard data are most reliable when they are matched with video footage to validate impact conditions and kinematics and to identify potential signs or symptoms of SRC, suggesting that a combined approach, as described herein for Cases 2 and 3, is best used whenever possible. A caveat to this, however, is that consideration needs to be given to particular camera angles when using limited video data, and whether linear or angular velocities predominate. Using both mouthguard data and video footage to identify the impact location, kinematics and the surface involved offers the most comprehensive and accurate information when modelling brain kinematics. Furthermore, research efforts towards a standardized diagnostic approach for SRC will aid the generation of “thresholds” for injury. Clinical studies which encompass objective and sensitive measures of SRC and which are adequately powered across a prospective controlled population of interest are needed.

In terms of the brain tissue injury thresholds shown in Table 17, and those which have more recently emerged as measures of brain injury for SRC, the MPS, strain rate and the cumulative damage measure (CSDM) are apparent in much of the literature. Correlation of mechanical strain or strain rate predictions in computational modelling simulations of injury has been demonstrated with neuroimaging or histopathology measures in both preclinical and clinical studies [30,73,74], with a more recent study examining the link between strain, strain rate and indicators of long-term neurological disorders [75]. A recent review paper [76] which examined mechanical characterisation of human brain tissue at conditions relevant to TBI showed that levels of 20% strain or greater at strain rates of 10/s or greater is a reasonable minimum threshold for producing injury to the brain, which are relevant findings for computational studies of head injury events [77,78,79]. An excerpt from that review paper on head injury metrics [76] expresses matters thus:

*“As new head injury metrics are developed with improved understanding of head impact tolerance in different populations and different impact environments, effective evaluation of those metrics will be crucial to determining their usability. Head injury metrics should be evaluated against real-world datasets that lack sampling bias. These datasets would include actual injury rates from a population, and head impact data from both high- and low-risk individuals to capture a complete exposure profile”.*

From our simulation predictions, and as discussed above for each of the five real-world cases, wearing the N-Pro headguard showed a noticeable reduction in many of the parameters investigated for each of the real-world injury events. Some of the various thresholds for likelihood of injury presented in Table 17 have been included in Figure 11, Figure 12 and Figure 13 since they are representative of prior work that has been performed to propose injury tolerances for head impact kinematics and brain tissue responses, the limitations of which are discussed herein. In terms of repeat impact exposure associated with multiple impacts that are either sub-concussive or concussive, it can be reasoned that continuous reductions in the kinematics and tissue deformations, such as predicted here when wearing the N-Pro headguard, would provide benefits to athletes subject to a high impact exposure.

The use of neuroimaging and of protein-based blood biomarkers to quantify the effects of repeated head impact exposure on brain microstructure and metabolism have offered promising research findings in recent years [27,28,29,30,31,32,33]. Using such evidence in clinically informed computational modelling predictions of head impact events would allow for thresholds or tolerances for SRC to be better quantified for a specific population and improvements in protective equipment to be made. A recent study into American football examined the links between repeated impact exposure, brain strain kinematics and a neurological biomarker [80,81]. For rugby, the World Rugby Headgear Innovation trial framework [68] offers manufacturers a unique opportunity to prospectively examine the effectiveness of their products in a real-world setting using objective measures with independent oversight from research experts in neurotrauma, neuroimaging, neurocognitive testing and biological biomarker analysis. Combining FE and computational modelling tools with such clinical datasets would add significant improvements to both risk prediction and development of protective measures for SRC.

### 4.7. Data Collection Methods

When comparing the two data collection methods used in this present study, several parameters and variables influence their respective effectiveness. Instrumented mouthguard data demonstrate greater reliability and accuracy, as their sensors provide direct inertial kinematic measurements. In contrast, video data are generally more readily available from at least one single camera angle, although this can be less accurate in estimating three-dimensional impact kinematics, particularly in the absence of three orthogonal camera angles.

Both Cases 2 and 3 used video footage and instrumented mouthguard data. In Case 2, the linear velocity components and their resultant values derived from the mouthguard and video data were in close agreement. However, the resultant angular velocity estimated from the video data was far greater than that estimated by the mouthguard, with the largest component of velocity being observed as the z-component (i.e., directed downward towards the ground). For cases without the headguard, the predicted peak levels of strain (MPS), von Mises stress (VMS), and linear acceleration (PLA) using either video or instrumented mouthguard kinematic data were within approximately 10% of each other as shown in Table 10 and Figure 11, Figure 12 and Figure 13.

Case 3, on the other hand, revealed more significant differences, with the instrumented mouthguard data providing more reliable measurements compared to video data and Kinovea. The resultant linear velocity from the mouthguard was more than twice as large as that estimated from video data. While the x and z components of linear velocity were consistent between the two methods, the y-component differed substantially. Similarly, the angular velocity measured by the mouthguard was significantly higher than that estimated from Kinovea. The IMM data are likely to be more accurate than the Kinovea data, as has been reported previously elsewhere [60]. Additionally, when analysing a head impact collision using Kinovea, it is highly desirable to have three or four separate or orthogonal viewpoints of the impact event [82]. Several factors contribute to errors associated with the video data collection method, including shifts in camera angle during impact, blurred or obscured footage, the lack of multiple camera perspectives, and the absence of clear, distinguishable head markers for tracking.

### 4.8. Limitations

There are various limitations to using the finite element analysis method, which is an approximate technique, when seeking to estimate the stresses and strains within neural tissue that are associated with an impact event. The most recent computational models (e.g., [40,47,48,50,59,73,75,79,83,84,85]) are generally more biofidelic than earlier generation models such as that used in the present study. As such, they are likely to provide more accurate estimates for stresses and strains, particularly in small anatomical regions such as the sulci, or in deep brain structures which are not explicitly modelled in this present study. Properly accounting for the constitutive behaviour of cerebrospinal fluid, for dynamic fluid/solid interactions, and for the rate dependent dynamic properties of various neural tissue that include oriented axonal fibre tracts tends to require more sophisticated, computationally intensive models. The advantage of such sophisticated models is that they can better predict the stresses and strains in some of the smaller brain regions, and these can be slightly greater or less than what is predicted using coarser and less sophisticated models [47,50,73]. This is important when seeking to provide quantitative estimates of stresses and strains that would be compared against either assumed or actual thresholds for concussion that would be used subsequently to inform clinical judgements regarding the severity of a particular impact event. However, the principal objective of this present study has been to compare the likely levels of impact attenuation associated with wearing or not wearing a headguard. Using more sophisticated computational models might indeed provide more accurate estimates of the stresses and strains within a player’s brain but they would also show similar levels of attenuation due to the wearing of a headguard.

The use of instrumented mouthguard data is a significant advance in the state of the art in head impact biomechanics. However, there are some caveats in respect of various aspects of this technology, particularly when it is used without having due regard to the visual data that is often evident on video footage. Consider, for example, the segmentation shown in Figure 2, which is associated with Prevent Biometrics’ mouthguard. These appear to be defined in a rather arbitrary, albeit convenient, manner which does not align with particular anatomical regions. A more refined segmentation, such as illustrated in Figure 1, would represent physical reality better, and consequently using the kinematic data to infer stresses and strains within the brain tissue from a finite element analysis such as performed in the present work would be more accurate. Whether such a more refined segmentation is computationally affordable with the hardware and software of the instrumented mouthguard is difficult to say, although this is likely to be an issue that will become easier to address as this technology develops further. Similarly, this study has not considered any effects that might be associated with any cumulative errors in using mouthguard sensors. If that is an issue of importance, it is likely to be addressed in any subsequent designs of mouthguards and sensor technology.

## 5. Conclusions

A principal purpose of this study was to evaluate the effectiveness of the N-Pro headguard as a protective device against head impacts for rugby players. This was accomplished by computationally simulating five real-world head impact scenarios using a FE head model, without and with a corresponding headguard model, during impacts against non-compliant surfaces. Additionally, two data acquisition methods were compared for capturing impact kinematics. The results demonstrate clearly that the N-Pro headguard reduces peak resultant head accelerations and brain tissue responses in all simulated cases, resulting in a reduction of typically 70% in peak linear accelerations. Depending on the impact location, the corresponding reductions in peak stresses and strain when wearing a headguard can also be as much as 70%, although not consistently so. In some cases, especially for impacts similar to Case 2 (Top Rear location), there may be negligible reduction in the stresses and strains. Notwithstanding this caveat, the results support the claims made by N-Pro regarding the increased protection against head impacts for players who wear their product, in comparison to those who do not wear any protection.

Additionally, the study allowed for an evaluation of the advantages and limitations of two methods for gathering impact kinematics. The use of wearable sensors such as instrumented mouthguards to objectively gather accurate measures of kinematic data is a tool that has gained significant attraction in the past few years. While it has significant potential for gathering accident information in real-time as compared to other methods such as motion tracking through video footage, it does come with its limitations. One of the major limitations in rugby impact reconstructions is that it does not provide a detailed description of the accident, which can limit the ability to fully understand the dynamics of the impact and accurately assess the potential risk of injury. Therefore, it is recommended to use video footage and analysis as a complement to the instrumented mouthguard data.

The relatively small sample size of head impact cases simulated in this present study limits the conclusions that can be drawn from the results. However, the results of the simulations do offer preliminary findings which suggest that the N-Pro headguard is effective in attenuating head impacts against a rigid, non-compliant surface, which may be applicable to many cases in contact sports. The primary condition under consideration in our simulations is impact against a rigid, non-compliant surface. While this controlled scenario is valuable for investigating the performance of the headguard without also having to account for many unknown variables, it will not fully represent the diverse range of conditions that are encountered in real-world sporting events. Contact sports involve multiple variables, including different playing surfaces and impact angles, and these have purposely not been considered in this present study. While this approach allows us to assess the effectiveness of the N-Pro headguard in its most conservative scenario, it is essential to acknowledge that real-world head impacts often involve a degree of surface compliance, which serves to absorb and dissipate some of the kinetic energy instead of all the impact energy being transmitted to and absorbed by a player’s head and their headguard. The predicted accelerations, stresses and strains of the present simulations are likely to be lower if ground compliance were to be considered explicitly. Furthermore, the results suggest that a large reduction in rotational and linear accelerations of the head does not necessarily translate to large reductions in brain tissue responses, i.e., Von Mises stresses and maximum principal strains. Hence, the overall results may provide an insight into the added benefits of using a variety of test methods, such as FE modelling, when determining the protective capabilities of sports headguards.

## Figures and Tables

**Table 1:** Descriptions of three real-world rugby head impact events used to extract impact velocities and locations implementing frame-by-frame video analysis.

Case ID	Impact Event Description	Video Web Link
1	Player landed on the back of head following aerial collision. Received an on-field medical exam and after passing HIA, continued playing.	https://www.youtube.com/watch?v=XVbfgka6XkE (accessed on 1 September 2024)
2	Player tackled with impact collision to top of head and opponent’s shoulder. Did not receive on-field medical exam, continued playing. Impact occurs at 16.33–16:34 in video.	https://www.youtube.com/watch?v=p9SsPuGrU1Q (accessed on 1 September 2024)
3	Player landed on the back of head following top of head collision with opposition torso. Did not receive on-field medical exam, continued playing. Impact occurs at 13.02–13:06 in video.	https://www.youtube.com/watch?v=FFC8Pn1HyRo (accessed on 1 September 2024)

**Table 2:** Impact location, peak linear and angular acceleration (PLA, PAA), peak linear and angular velocity (PLV, PAV), maximum principal strain (MPS), and brain region affected as reported by Prevent Biometrics for each case.

Case ID	Location	PLA (g)	PAA (rad/s^2^)	PLV (m/s)	PAV (rad/s)
2	Top Rear	69.2	1276	1.9	7.4
3	Top Rear	37.2	2031	2.3	18.5
4	Front High	55.4	6807	1.5	19.4
5	Left High	55.3	5547	2.7	30.8

**Table 3:** UCDBTM and N-Pro Headguard material properties [38,64].

Material	Young’s Modulus (MPa)	Poisson’s Ratio	Density (kg/m^3^)
Scalp	16.7	0.42	1000
Cortical Bone	15,000	0.22	2000
Trabecular Bone	1000	0.24	1300
Dura	31.5	0.45	1130
Pia	11.5	0.45	1130
Falx and Tentorium	31.5	0.45	1130
Brain	Hyper Elastic	0.4999	1040
Facial Bone	5540	0.22	2100
CSF	0.15	0.49989	1000
Grey Matter	Viscoelastic	0.49	1040
White Matter	Viscoelastic	0.49	1040
N-Pro Headguard	5	0.3	249.886

**Table 4:** UCDBTM Brain material properties [38,64].

Material	Prony Parameters				Mooney-Rivlin Parameters		
	g_1_	g_2_	τ_1_	τ_2_	C10	C01	D1
Brain	0.527298	0.30344	0.008	0.145	3653.5	4059.44	9.46 × 10^−10^

**Table 5:** Peak linear acceleration (PLA) comparison.

Impact Zone	Experimentally Recorded PLA (g)	Simulated PLA at CoM (g)	Average Simulated PLA (g)
Crown	140	95	93.21
Forehead	95	135	122.53
Temple	130	162	157.75

**Table 6:** x, y, z components and resultant magnitudes of the linear and rotational velocities, for each real-world impact case, used as input into the UCDBTM.

Case	Data Source	Linear Velocity [m/s]				Rotational Velocity [rad/s]			
		Resultant	x	y	z	Resultant	x	y	z
1	Video Analysis	4.97	0	−4.58	−1.93	0.81	−0.70	0.41	−0.02
2	Video Analysis	2.335	−1.37	−0.34	−1.86	8.79	−0.77	0.88	−8.71
	Prevent IMM	2.348	−1.63	0.174	−1.681	3.67	−0.165	3.577	−0.806
3	Video Analysis	0.87	−0.3	0.41	−0.71	7.47	−1.37	0.34	7.33
	Prevent IMM	2.316	−0.298	2.137	−0.841	18.519	16.94	5.907	4.606
4	Prevent IMM	1.630	1.22	0.98	−0.46	18.500	4.05	−4.17	17.57
5	Prevent IMM	2.720	−0.52	−2.66	−0.22	29.770	−26.34	6.7	12.15

**Table 7:** Case 1 linear and rotational velocities used as input for the UCDBTM. The coordinate system is centred in the centre of gravity of the UCDBTM, with x pointing posterior to anterior, y left to right and z downwards.

Location	Linear Velocity (m/s)				Rotational Velocity (rad/s)			
	Resultant	x	y	z	Resultant	x	y	z
L3	4.97	0.00	−4.58	−1.93	0.81	−0.70	0.41	−0.02

**Table 8:** Predicted peak resultant head kinematics and brain tissue response for Case 1.

Parameter	Unprotected	Protected	% Reduction
Von Mises Stress (kPa)	19.52	14.12	27.66
Max. Principal Strain (%)	35.70	28.35	20.59
Peak Linear Acceleration (g)	663.66	183.78	72.31

**Table 9:** Case 2 linear and rotational velocities of both IMM and Kinovea data used as input for the UCDBTM. The coordinate system is centred in the centre of gravity of the UCDBTM, with x pointing posterior to anterior, y left to right and z downwards.

Location	Collection Method	Linear Velocity (m/s)				Rotational Velocity (rad/s)			
		Resultant	x	y	z	Resultant	x	y	z
Top Rear	Kinovea	2.335	−1.37	−0.34	−1.86	8.79	−0.77	0.88	−8.71
	Mouthguard	2.348	−1.63	0.174	−1.681	3.67	−0.165	3.577	−0.806

**Table 10:** Predicted peak resultant head kinematics and brain tissue response for Case 2 using data collected by Kinovea and Prevent IMM.

Collection Method	Parameter	Unprotected	Protected	% Reduction
Kinovea	Von Mises Stress (kPa)	7.98	7.56	5.25
	Max. Principal Strain (%)	15.15	14.78	2.44
	Peak Linear Acceleration (g)	442.46	102.62	76.81
Instrumented Mouthguard (IMM)	Von Mises Stress (kPa)	7.09	6.97	1.69
	Max. Principal Strain (%)	13.42	12.78	4.77
	Peak Linear Acceleration (g)	449.86	99.38	77.91

**Table 11:** Case 3 linear and rotational velocities of both IMM and Kinovea data used as input for the UCDBTM. The coordinate system is centred in the centre of gravity of the UCDBTM, with x pointing posterior to anterior, y left to right and z downwards.

Location	Collection Method	Linear Velocity (m/s)				Rotational Velocity (rad/s)			
		Resultant	x	y	z	Resultant	x	y	z
Top Rear	Kinovea	0.87	−0.3	0.41	−0.71	7.47	−1.37	0.34	7.33
	Mouthguard	2.316	−0.298	2.137	−0.841	18.519	16.937	5.907	4.606

**Table 12:** Predicted peak resultant head kinematics and brain tissue response for Case 3 using data collected using Kinovea and Prevent IMM.

Collection Method	Parameter	Unprotected	Protected	% Reduction
Kinovea	Von Mises Stress (kPa)	2.10	1.83	12.88
	Max. Principal Strain (%)	4.59	4.14	9.79
	Peak Linear Acceleration (g)	92.69	24.28	73.80
Instrumented Mouthguard (IMM)	Von Mises Stress (kPa)	6.80	3.99	41.32
	Max. Principal Strain (%)	13.52	9.74	27.96
	Peak Linear Acceleration (g)	127.71	26.61	79.16

**Table 13:** Case 4 linear and rotational velocities used as input for the UCDBTM. The coordinate system is centred in the centre of gravity of the UCDBTM, with x pointing posterior to anterior, y left to right and z downwards.

Location	Linear Velocity (m/s)				Rotational Velocity (rad/s)			
	Resultant	x	y	z	Resultant	x	y	z
Front High	1.630	1.22	0.98	−0.46	18.500	4.05	−4.17	17.57

**Table 14:** Predicted peak resultant head kinematics and brain tissue response for Case 4 using data collected by Instrumented Mouthguard (IMM).

Collection Method	Parameter	Unprotected	Protected	% Reduction
Instrumented Mouthguard (IMM)	Von Mises Stress (kPa)	17.58	4.13	76.51
	Max. Principal Strain (%)	32.56	8.67	73.36
	Peak Linear Acceleration (g)	226.85	64.31	71.65

**Table 15:** Case 5 linear and rotational velocities used as input for the UCDBTM. The coordinate system is centred in the centre of gravity of the UCDBTM, with x pointing posterior to anterior, y left to right and z downwards.

Location	Linear Velocity (m/s)				Rotational Velocity (rad/s)			
	Resultant	x	y	z	Resultant	x	y	z
Left High	2.720	−0.52	−2.66	−0.22	29.770	−26.34	6.7	12.15

**Table 16:** Predicted peak resultant head kinematics and brain tissue response for Case 5 using data collected by Instrumented Mouthguard (IMM).

Collection Method	Parameter	Unprotected	Protected	% Reduction
Instrumented Mouthguard (IMM)	Von Mises Stress (kPa)	16.90	13.21	21.83
	Max. Principal Strain (%)	31.99	26.26	40.96
	Peak Linear Acceleration (g)	824.61	284.13	65.54

**Table 17:** Proposed thresholds for SRC injury risk from several studies into real-world impact events.

Reference	Sport	Sample Size/Number of Injury Events	Threshold/Injury Risk	VMS (kPa)	MPS (%)	PLA (g)	PRA (rad/s^2^)
[9]	American football	Head-to-head collisions involving 24 players in 12 cases (at least 12 players had confirmed concussion)	50% chance; midbrain	7.8	19	82	5900
			80% chance; midbrain	10	24	106	7900
[34]	AFL and rugby	27 cases of concussive head impacts	Mean peak values reported for injuries	-	-	103	8022
[35,36]	American football, AFL and rugby	27 cases of concussion (same cases as Frechede and McIntosh), and 13 non-injurious cases	50% chance; thalamus	2.24	13	-	-
			50% chance; corpus callosum	3.51	15	-	-
			50% chance; white matter	-	26	-	-
			50% chance; midbrain	15.1	-	-	-
[37]	Equestrian	25 concussive and 25 non-concussive impacts	50% risk	6.6	24	59	2700

## Data Availability

Data available on request from the corresponding author.

## Appendix A

**Table A1:** Orientation of the UCDBTM at impact for the four real-world reconstructions.

Case	Unprotected	Protected
1		
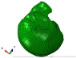
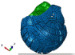
2		
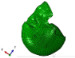
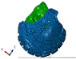
3		
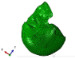
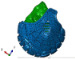
4		
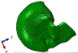
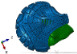
5	****	****
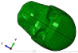
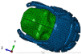

**Table A2:** Maximum principal strain distribution over the brain for the five unprotected and five protected cases. The impact locations are circled in red.

Case	Unprotected	Protected
1 Kinovea		
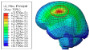
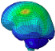
2 Kinovea		
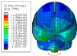
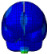
2 Prevent IMM		****
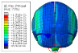
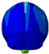
3 Kinovea		
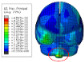
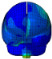
3 Prevent IMM		****
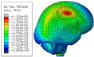
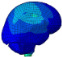
4 Prevent IMM		****
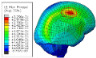
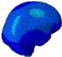
5 Prevent IMM		
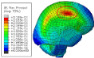
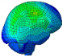

**Table A3:** Von Mise’s stress distribution over the brain for the five unprotected and five protected cases. The impact locations are circled in red.

Case	Unprotected	Protected
1 Kinovea	****	****
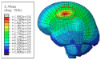
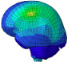
2 Kinovea	****	****
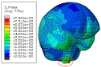
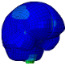
2 Prevent IMM	****	****
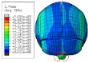
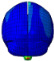
3 Kinovea	****	****
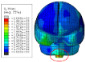
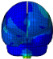
3 Prevent IMM	****	****
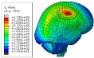
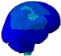
4 Prevent IMM	****	****
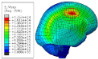
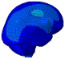
5 Prevent IMM	****	****
